# Recent Advances in Mesoporous Silica Nanoparticles Delivering siRNA for Cancer Treatment

**DOI:** 10.3390/pharmaceutics15102483

**Published:** 2023-10-17

**Authors:** Xiaowei Xie, Tianxiang Yue, Wenting Gu, WeiYi Cheng, Li He, WeiYe Ren, Fanzhu Li, Ji-Gang Piao

**Affiliations:** School of Pharmaceutical Sciences, Zhejiang Chinese Medical University, Hangzhou 310053, China; xiaoweixie6@163.com (X.X.); alivedx808@163.com (T.Y.); wentingtina@foxmail.com (W.G.); cwy9999@foxmail.com (W.C.); hl18063069708@163.com (L.H.); 19858117356@sina.com (W.R.)

**Keywords:** siRNA delivery, mesoporous silica nanoparticles, RNAi therapeutics, cancer treatment, gene therapy

## Abstract

Silencing genes using small interfering (si) RNA is a promising strategy for treating cancer. However, the curative effect of siRNA is severely constrained by low serum stability and cell membrane permeability. Therefore, improving the delivery efficiency of siRNA for cancer treatment is a research hotspot. Recently, mesoporous silica nanoparticles (MSNs) have emerged as bright delivery vehicles for nucleic acid drugs. A comprehensive understanding of the design of MSN-based vectors is crucial for the application of siRNA in cancer therapy. We discuss several surface-functionalized MSNs’ advancements as effective siRNA delivery vehicles in this paper. The advantages of using MSNs for siRNA loading regarding considerations of different shapes, various options for surface functionalization, and customizable pore sizes are highlighted. We discuss the recent investigations into strategies that efficiently improve cellular uptake, facilitate endosomal escape, and promote cargo dissociation from the MSNs for enhanced intracellular siRNA delivery. Also, particular attention was paid to the exciting progress made by combining RNAi with other therapies to improve cancer therapeutic outcomes.

## 1. Introduction

The Food and Drug Administration (FDA) approved the first RNA interference (RNAi) therapeutic in 2018, Patisiran [1], a lipid nanoparticle-coated small interfering (si)RNA, thus translating fundamental research into clinical practice. This milestone event signified a breakthrough in the combinatorial use of RNAi and nanotechnology to treat diseases, ushering in a new era of gene therapy. RNA therapies aim to alter gene expression or generate therapeutic proteins, making them appropriate for pathologies with known genetic targets, including infectious diseases, immunological diseases, and cancer [2,3]. Due to its high malignancy, low survival rate, poor prognosis, and ease of recurrence, cancer poses a significant risk to human society. Currently, surgery is the mainstay for cancer treatment in clinical settings, with chemotherapy as a backup. However, the therapeutic efficacy is insufficient because of the toxic side effects of chemotherapeutic agents, poor selectivity, and drug resistance. In addition to chemotherapeutic drugs, therapeutic RNAs such as miRNAs and siRNAs can be delivered by appropriate carriers into target cells to interact with specific genes and up/downregulate their expression [4,5,6]. RNA therapies have consequently emerged as a promising therapeutic option for cancer.

siRNAs, with a length of 20 to 25 nucleotides, can bind to specific complementary mRNA pairs, thus triggering mRNA degradation through the RNA-induced silencing complex (RISC) to exert therapeutic effects. Currently, RNAi therapies can be classified based on the distinct targets of selected therapeutic genes, including reversing the drug resistance of tumor cells, promoting apoptosis, inhibiting angiogenesis, or acting synergistically with immunotherapy. Among them, reversing the drug resistance of tumor cells by altering the expression of resistance proteins through gene silencing is a frequent therapeutic strategy. The gene regulation of apoptosis-related proteins is an attainable antitumor approach to enhance drug-induced apoptosis and improve cytotoxicity. Moreover, overexpression of the Vascular Endothelial Growth Factor (VEGF) in tumor tissues drives angiogenesis and blocks the VEGF signaling pathway, which can be diminished by RNAi to prevent tumor growth [7,8]. siRNA-based gene therapy for the knocking down or downregulating immune checkpoint proteins (e.g., PD-L1 and STAT3), “don’t eat me” signal (e.g., CD47), and anti-inflammatory cytokines (e.g., TGF-β) induces antitumor immune responses and significantly inhibits tumor growth in vivo [9,10,11,12]. However, delivering RNA remains a challenge due to short blood circulation, poor cellular uptake, susceptibility to nuclease-mediated degradation, and low transfection rates [13,14]. As a result, there is an urgent need to develop a safe and effective delivery method for siRNA loads.

The delivery of siRNAs is commonly performed through viral and nonviral vectors [2]. Due to their great effectiveness, viral vectors—primarily lentivirus, adenovirus, and adeno-associated viruses (AAVs)—are frequently used for transfection [15]. Nevertheless, immunogenetic responses, inaccurate viral-genome integration, the inability to redose, and the high cost of vector production pose hurdles to their application [16,17,18,19]. On the other hand, nonviral vectors have their own advantages such as enhanced permeability and retention (EPR) effects, better pharmacokinetic and pharmacodynamic properties, reduced hepatic accumulation, and lower toxicity [20,21]. The most common nonviral vectors include polymeric nanoparticles, micelles, liposomes, inorganic nanoparticles, etc. [22,23,24]. Polymeric nanoparticles are processed with good biocompatibility and biodegradability but easily combine with negatively charged nonspecific cells or proteins. Micelles show the advantages of a long retention time in the body and good tissue penetration, but there are shortcomings in drug leakage and burst release.

Mesoporous silica nanoparticles (MSNs) are being considered as a promising delivery vehicle for a variety of biomedical applications, owing to their high biocompatibility [25,26]. MSNs have a distinctive structure and large specific surface area, which allows for efficient drug delivery. Additionally, the chemical functional groups present on their surfaces enable the introduction of other functionalities. In 2016, researchers investigated the usage of MSNs to codeliver anticancer drugs and siRNA for the treatment of tumors and discussed the potential of MSNs and combination therapy to enhance chemotherapy results [27].

We searched for articles published in the last ten years that discuss the use of MSNs as delivery vehicles for cancer treatment. Our review includes an introduction to the characteristics of MSNs and how they are efficiently loaded with siRNA for precise delivery to tumor cells. We also provide an overview of cancer-treatment strategies that utilize siRNA-loaded MSNs. In conclusion, we discuss the potential uses of siRNA-loaded MSNs in tumor diagnosis and therapy.

## 2. MSNs as a Vector of siRNAs

### 2.1. The Characteristics of MSNs

MSNs have a unique structure that includes a silica matrix with organized pores, providing a large surface area, enormous pore volume, and variably sized pores with a narrow distribution [28] that allow them to hold large quantities of drugs. The physical features, including the morphology, size, and aspect ratio, can impact cellular phagocytosis and other biological processes [29,30]. Owing to the simplicity of their synthesis and well-established silanol chemistry, MSNs can be subjected to a wide range of physical and chemical alterations to tailor them for specific purposes [31,32]. On the one hand, different shapes and aspect ratios of MSNs can be designed to acquire a larger surface area and higher loading efficiency. On the other hand, MSNs can be designed to load various drugs, both hydrophobic and hydrophilic, efficiently, via modifying the pore surface chemistry and sizes. Biocompatibility is a crucial element in determining the compatibility of materials with the human body. Silicon or silicon substances are commonly found in food and ingested by humans, and MSNs are believed to have good biocompatibility in vivo, based on FDA reports [33,34,35]. Overall, MSNs offer great potential for drug delivery due to their unique structure and versatility in design [36].

### 2.2. Therapeutic siRNA Payloads

**Figure 1:**
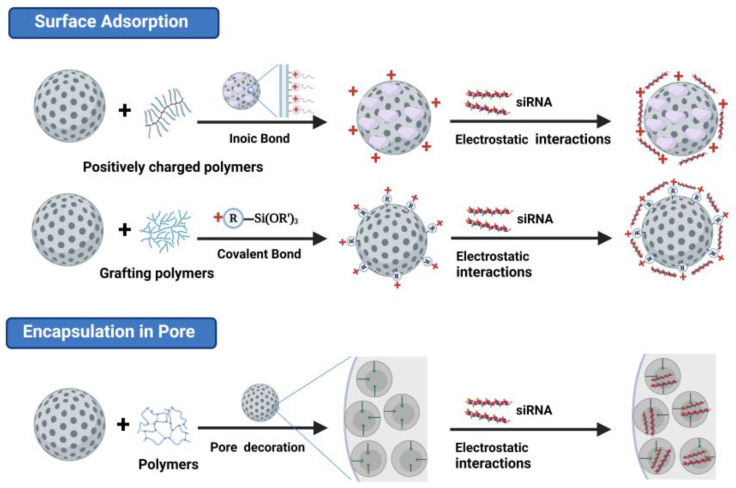
Mechanisms of small interfering (si)-RNA-loaded mesoporous silica nanoparticles (MSNs) after modification.

Since MSNs are one of the prominent nanocarriers for cancer therapy, the synthesis strategies of MSNs have been comprehensively reviewed [37]. To modify the surface of MSNs, both noncovalent and covalent modifications can be performed due to their unique physicochemical and physiological properties. Cationic molecules are usually employed to coat the surface of MSNs through noncovalent strategies to enable electrostatic interactions with nucleic acids when preparing siRNA-loaded MSNs [38]. The siRNAs are deposited onto MSNs via electrostatic and hydrophobic interactions and are quickly released into cells upon delivery. Polyamine polymers and cationic dendrimers are commonly used for siRNA delivery. In addition, a class of polymer materials, cationic peptides, can also serve as nucleotide carriers if appropriately functionalized [39,40]. Moreover, in contrast to other nanomaterials, MSNs’ distinctive pore structure enables the loading of various cargoes inside the pores [41]. MSNs with vast pores (>10 nm) have also been developed by some research groups to broaden the application of MSNs in nucleic acid delivery [42,43]. Additionally, the complexes’ siRNA/MSN ratio is typically calculated and tuned by evaluating the complexation and delivery efficiency of MSNs towards siRNA [44]. Two main strategies are currently employed for siRNA delivery using MSNs, namely the surface loading of siRNAs onto MSNs and containing siRNAs inside the pores of MSNs (Figure 1).

#### 2.2.1. Loading siRNAs onto the Surface of MSNs

Nucleic acids, bearing negative charges from phosphate groups, cannot directly bind to bare MSNs with silicon hydroxy groups. Typically, positively charged polymers like poly-l-lysine (PLL) or polyethyleneimine (PEI) are added to MSNs to facilitate the binding of negatively charged nucleic acids via electrostatic interactions [45,46]. Researchers have coated MSNs with varying molecular weights of PEI to create a positively charged surface and then loaded siRNAs. In vitro, investigations suggest that MSNs can efficiently shield siRNA from degradation and induce gene silencing without altering the siRNA structure. A simple yet efficient delivery method based on PEI-coated MSNs was presented to evaluate the release kinetics of siGLO [44]. The noncovalent attachment of PEI to MSNs both facilitates the cellular uptake of MSNPs and creates suitable conditions for siRNA conjugation. The maintenance of good cellular uptake and a good transfection efficiency was possible while lowering or even removing cationic cytotoxicity by experimenting with various polymer molecular weights. For a more-effective siRNA delivery, Rafatosadat and coworkers simultaneously treated MSN with Zn^2+^ and PEI. The MSNs were synthesized by a traditional hydrothermal method and modified with zinc ions [47]. Owing to the vast surface charge density of Zn^2+^ and the cationic nature, PEI-Zn-MSN showed a higher zeta potential and consequently a better siRNA adsorption capacity than PEI-MSN alone. Additionally, grafting a layer of polyethylene glycol (PEG) onto the surface of PEI-Zn-MSN can enhance its release profile without causing cytotoxicity.

The cationic polymer coated on the surface of MSNs by electrostatic binding comes off after i.v. administration due to competing interactions with charged macromolecules in the bodily fluid [46]. MSNs with surface silanol groups are not optimal for direct siRNA capture. Modification with amines, carboxylic acids, and thiols is possible. Covalent bonding is an alternate way of anchoring the polycationic polymer. Second-generation (G2) polyamidoamines (PAMAMs) can covalently attach to the surface of isocyanatopropyl-functionalized MSNs (ICP-MSNs) via a urea linkage [48]. Grafting poly (dimethylaminoethyl methacrylate) PDMAEMA onto the surface of aminated MSN not only allowed it to highly react with nucleic acid and protect siRNA from enzymatic destruction but also had a vast electrostatic affinity with the cell membrane, increasing MSN cell absorption [49]. Nonpolymeric coatings such as triethoxysilane (APTES) provided positively charged amine groups. Because it contains both silane and amino groups that can pair with the silanol groups of MSNs, APTES is commonly employed for the amine functionalization of MSNs [50,51]. Covalent grafting using APTES via glutaraldehyde as the linker functionalized MSNs with chitosan [52]. An amine-rich surface coating was created by using APTES and grafted chitosan, permitting the electrostatic loading of both MTX and siRNAs. In vitro studies on cell uptake, cytotoxicity, and STAT3 expression in MCF7 cells reveal suppressed cellular division and proliferation as well as lowered STAT3 expression at the mRNA and protein levels.

#### 2.2.2. Containing siRNAs Inside the Pores of MSNs

Compared to other nanomaterials, MSNs have a unique pore structure that enables the loading of various cargos inside the pores [53]. The mesoporous structure of MSNs to encapsulate nucleic acid can significantly improve their loading rate; their surface can be further altered, which not only prevents nucleases from destroying nucleic acid but also significantly reduces particle aggregation owing to bridging. However, because of RNA’s vast sizes, the loading of nucleic acids into narrow pores is typically impossible. To broaden the applicability of siRNA delivery, several scholars have created MSNs with enormous pores capable of encapsulating siRNAs, which improved the loading and delivery efficiency [54,55,56,57,58,59,60,61] (Table 1).

Na et al. compared the application of two types of MSNs, MSN2 (pore radius = 1.05 nm) and MSN23 (pore radius = 11.5 nm) as siRNA delivery systems [51]. Electrostatic attraction is anticipated to be one of the primary forces guiding the loading of siRNA onto MSNs as a result of the opposite charges of siRNA and amine-functionalized MSNs. The zeta potential is a characteristic of MSNs that screens the electrostatic potential in the dispersing medium and is inversely proportional to the MSNs’ effective surface charge density. MSN2 pores have a higher potential than the outside surface has, and the relative difference between the two potentials gets larger as the pore size gets smaller. Although the field towards the pores is strong, the siRNA molecules (the gyration radius ≈ 2.0 nm) are larger than the pore size, preventing siRNA from entering the pores. The other way around, for MSN23 with a pore size larger than siRNA, siRNA can enter the pores without restriction and stick to the pore walls. Even if some siRNA is deposited on the MSNs’ outside, the majority of siRNA is likely to be deposited inside the pores. Owing to the difference in pore size, the release of siRNA from the two types of MSNs also starts at different points.

The utilization of siRNA transfection technology based on MSN-based devices has provided a new approach for the development of multifunctional delivery vectors with relative hypotoxicity and excellent efficiency. Therapeutic siRNAs can be loaded onto the surface of MSNs or inside their pores. However, it is challenging to determine which strategy between these two can provide better results for widespread applications. On the one hand, a number of antitumor applications can considerably benefit from the use of MSNs’ wide pores. For instance, employing molecules to encase and cover other functional molecules inside the pores can result in tumor-specific “on-demand” release [62]. Additionally, the successful construction of intelligent siRNA delivery systems has been accomplished through biomimetic strategies, enabling the targeted delivery of siRNA within tumor cells [63]. However, the complexity and uncertainty of the loading process limit the widespread application of this transfection method, while the intramesoporous loading of siRNA requires excellent preservation of the loaded siRNAs. On the other hand, the surface deposition of siRNA on MSNs is a straightforward, controllable, and measurable process, allowing for better control of the loading and development of various intelligent delivery strategies. This is of great interest for the purpose of siRNA-based synergistic therapy and the diagnosis of tumors.

**Table 1:** Particle and pore sizes in systems for delivery of siRNA using MSNs.

Drug-Delivery System	Size (nm)	Pore Size (nm)	Pore Volume (mL/g)	BET Surface Area (m^2^/g)	siRNA Type	RNA Capacity	Ref.
MSN_23_	200	23	0.97	395	siGFP	1.25 pmol/ug	[36]
Protocells	165	23–30	/	850	Cyclin-specific siRNA	~6 × 10^4^ siRNA per particle	[46]
M-MSNs	50	3.6	/	/	siVEGF and siEGFP	~27.5 μg/mg	[47]
ssCP-MSNs	100–150	10	0.63	159	siPLK1	/	[48]
PEI-Fe-LPMSN	200	4.6	0.33	217	siPLK1	/	[49]
MSNPs-PLL	250	4	/	/	TGFβR-1 siRNA	3.13 wt%	[51]
MP-1	150	4.7	0.75	670	STAT3 siRNA	380 µg per mg MSN	[52]
CMSN-A	80–110	21	1.42	481	FAN-siRNA	148 μg siRNA/mg	[53]
MNC@LPMSA	291	12	1.13	411	siPLK1 and siEGFP	2 wt%	[64]

## 3. Strategy to Achieve Efficient siRNA Delivery

**Figure 2:**
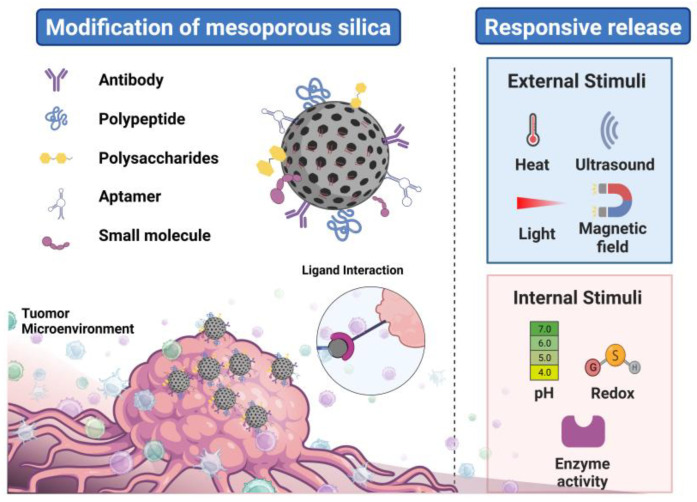
MSN-based active-targeting delivery and responsive release of siRNA for cancer RNA interference (RNAi) therapeutics.

MSN-mediated siRNA delivery offers distinct advantages. The unique modification on the surface of MSNs allows the complete delivery system to improve target cell recognition and enhance cellular uptake. MSNs with siRNA are endocytosed into the cytoplasm via endocytosis and escape from the endosome. Then, they precisely release siRNA under conditions of the internal and external activation of tumor cells, boosting the efficacy of siRNA-targeted therapy for cancers (Figure 2). We concentrate on the distinct mechanisms of MSNs as gene carriers as well as strategies for enhancing siRNA delivery effectiveness.

### 3.1. Enhancement of Cellular Uptake of MSNs

MSNs have gained widespread interest as vectors and are promising candidates for packaging siRNAs. However, achieving the controllability and accuracy of delivery remains a challenge. In the field of targeted delivery, biomarkers that are highly or specifically expressed at the target site are usually used as targets, and targeted molecules, antibodies, and polypeptides are used to achieve specific binding to these targets. According to this idea of receptor-specific binding, siRNA-loaded MSNs can directly attach to the appropriate receptor, which is highly expressed on the surface of tumor cells, when modified by ligands including the targeting peptide, antibody, aptamer, small molecule, and polysaccharide [65,66,67]. Subsequently, MSNs enter the cell through receptor–ligand-mediated endocytosis.

#### 3.1.1. MSNs Decorated with Peptides

In the MSNs’ delivery system, adding targeting peptides on their surface as ligands to selectively bind them to the corresponding receptors on the cell surface is a vital approach to achieving targeted transportation. Targeting peptides are a collection of linear or cyclic amino acids that bind to particular attaching sites on transmembrane receptors to specifically anchor vectors to target cells. These are generally composed of less than 50 amino acids. Compared to proteins, peptides with smaller molecular weights have several advantages, like more stability, resistance to environmental conditions, and ease of synthesis and conjugation. Currently, three main methods for screening tumor-targeting peptides are practiced: phage display technology, chemically synthesized peptide library screening, and computer simulation design based on tumor-specific targets.

Peptide-conjugated MSNs are popular for siRNA-targeting delivery. Some representative peptides are utilized for targeting tumor areas with an emphasis on cancers that are now of great interest and those that might benefit from more-effective siRNA delivery (e.g., breast cancer, primary liver cancer, and lung cancer). At present, the Arg-Gly-Asp (RGD) family of peptides is widely used, and the binding site is integrin αvβ3, which is overexpressed in tumor cells and tumor neovascular endothelial cells [68,69]. Thus, RGD-conjugated MSNs show enhanced selective endocytosis in cancer cells and angiogenic endothelial cells. The MSNs’ surface was covered with functionalized PEG chains (DSPE-PEG-DBCO) followed by the conjugation of iRGD by using copper-free click chemistry between azido-terminated iRGD and cyclooctyne-containing DSPE-PEG-DBCO. Wang and coworkers constructed an iRGD-modified lipid-coated MSN (iMSN) and utilized it for the codelivery of siRNA (siPlk1) and miRNA (miR-200c) [70]. N-terminal azido-functionalized iRGD peptide (N3-Ac (CRGDKGPDC)) was synthesized to enable deep tumor penetration. siPlk1 silenced the cell cycle mitotic regulator, Polo-like kinase 1 (PLK1), inhibited cell proliferation, and induced apoptosis. Despite having many advantages compared to other ligands, peptides face challenges in targeted drug delivery due to their high conformational flexibility (which may bring about limited receptor selectivity) and instability [71]. These limitations have become critical issues in the field, and any breakthrough in overcoming these drawbacks is urgently needed for the development of peptide-based targeted delivery vectors.

#### 3.1.2. MSNs Decorated with Antibodies

The antibody is a glycoprotein with a high affinity to receptors and a unique specificity to recognize receptors, which has been widely evaluated as a targeting ligand. Modified antibodies can accomplish the targeted delivery of drugs by antibody–ligand or antibody–receptor binding. The basic principle of actively targeted drugs constructed based on the antibody–receptor binding strategy is that certain receptors are highly expressed in tumor tissues. After binding to the surface membrane receptors of tumor cells, antibodies cause receptor-mediated endocytosis [72,73]. Therefore, the delivery system constructed by using RNA drugs coupled with corresponding antibodies can target tumor tissues.

A transmembrane glycoprotein with a coding molecular weight of 185 kDa, human epidermal growth factor receptor 2 (HER2), includes an intracellular tyrosine kinase catalytic region, a transmembrane region, and an extracellular ligand-binding region [74]. When the receptor anchors to the ligand, the ligand activates the receptor’s dimerization to form a dimer. HER2 undergoes dimerization and endocytosis to bind the receptor and ligand to cells. Trastuzumab (Herceptin) is a well-recognized humanized anti-HER2 monoclonal antibody that directly targets the extracellular ligand-binding region of HER2 and has been successfully applied to cure early-stage and metastatic HER2-overexpressing breast cancer [75]. Ngamcherdtrakul and coworkers prepared a new generation of siRNA delivery constructs in vivo with a 50 nm MSN core encapsulated with crosslinked polyethyleneimine-polyethylene glycol copolymers, carrying targets for human epidermal growth siRNA of the HER2 oncogene, coupled to trastuzumab [76]. A surface modification of 50 nm MSNPs was performed by using crosslinked PEI and PEG. The crosslinked PEI efficiently loaded negatively charged siRNA and facilitated intracellular escape through the proton sponge effect, while PEG protected siRNA from enzymatic degradation and nanoparticle uptake. A thiol-maleimide process was used to bind the antibody trastuzumab (T) to MSNP PEI-PEG. This construct was created to lengthen the siRNA in blood’s half-life, enhance the uptake of tumor-specific cells, and improve the siRNA knockdown effectiveness. They further designed a novel nanodelivery vehicle (T-siHER2-NP(DTX)) for the treatment of HER2+ breast cancer, namely a combination of taxane and HER2-targeting antibodies [77]. At the same dose, the efficacy of the drug-free counterpart (trastuzumab + docetaxel) was lower than that of T-siHER2-NP(DTX). Affinity and size play important roles in antibody-based targeted delivery strategies. While the tumor uptake of large molecules (full-length antibodies and antibody-conjugated nanoparticles) is primarily influenced by the EPR effect, the high retention of small molecules (single-chain antibodies) at the tumor site relies on their binding kinetics [78,79,80]. Another important aspect to take into account when choosing solid-tumor-targeting antibodies for therapeutic applications is antibody penetration, which is the uniform distribution of antibodies throughout the tumor. A high affinity and internalization are two factors that limit tumor penetration [81]. Wittrup’s group have documented that rapid internalization rates and degradation metabolism impede antibody penetration [82].

#### 3.1.3. MSNs Decorated with Aptamers

Aptamers are single-stranded DNA or RNA sequences that bind specifically to corresponding targets by forming a specific conformation [83]. Aptamers are mainly synthesized by the exponential enrichment ligand system evolution technique [84]. Aptamers are usually smaller in size, faster for synthesis, less costly, flexible, have low immunogenicity, and can be altered chemically in a variety of ways [85,86]. Additionally, many aptamers can internalize after anchoring to ligands on the cell surface, making them an effective offsider for precise delivery. AS1411, with a high affinity for nucleolin, has been employed to improve cells’ cancer-targeting ability [87]. A unique vehicle was created by Zhuang and colleagues to deliver doxorubicin (DOX) and antimiR-21, an oncogenic antagomir, simultaneously inside cells. A thiol-containing organosilane, 3-mercaptopropyltrimethoxysilane (MPTMS), was used to introduce -SH groups onto the surface of MSNs to obtain MSN-SH. Subsequently, MSN-SH was coincubated with DOX to allow its loading into the porous structure of MSN-SH. Finally, a thiol-functionalized oligonucleotide containing siTIE2 and an AS1411 aptamer was added to the solution and immobilized onto MSN-SH@Dox through a disulfide exchange reaction to block the mesopores. The authors modified the surface of MSNs with disulfide bonds to construct a suitable redox-responsive delivery system aimed at facilitating the intracellular capture and controlled release of Dox and siTIE2 for tumor cells. AS1411, through its interaction with nucleolin, undergoes a conformational change that allows for the release of multiple Dox molecules from MSNs, functioning as a nanogatekeeper. Due to limited toxicological information regarding nucleic acid aptamers, their potential toxicity and side effects and underlying mechanisms are not fully understood. High concentrations of nucleic acids may lead to potentially toxic reactions, including nonspecific tissue accumulation and nonspecific immune responses. In the future, it is important to assess the biocompatibility and safety of MSNs binding with aptamers for better clinical translation.

#### 3.1.4. MSNs Decorated with Small Molecule

Small molecules, i.e., molecules with a low molecular weight (usually less than 1000 Da), have attracted research attention for cancer targeting owing to their straightforward structure and inexpensive cost. As a targeting ligand, small molecules bind specifically to the receptors overexpressed in tumors and thus have been widely studied for their application in tumor-targeted therapy. Among these, folic acid (FA) stands out as the most up-and-coming targeting molecule due to its excellent affinity to the folate receptor.

Folate receptors (FRs), which are transmembrane glycoproteins linked by glycosylphospholipid acyl alcohols with a molecular weight 38–40 kDa, exist on most tumor cell surfaces and are rarely expressed in normal body cells [88]. FRs are highly expressed in ovarian cancer, brain cancer, kidney cancer, lung cancer, colon cancer, nasopharyngeal cancer, and other cancers [89]. FRs can transfer folate and folate drug complexes into tumor cells by endocytosis and further release drugs for therapeutic effects. Targeted antitumor drugs mediated by folate can improve the antitumor drug targeting and reduce the antitumor drug distribution in normal tissues, thereby attenuating adverse reactions [90]. Folate-modified MSNs show a selective enhancement of endocytosis in cancer cells to achieve the therapeutic effects of RNAi [65]. In this study, an FA-modified MSN nanocarrier was designed and synthesized. First, FA was covalently bound to the surface of MSNs via an amide bond that was vulnerable to acidity. Subsequently, siVEGF was loaded into the nanocarrier through electrostatic interactions. Furthermore, tumor-targeted MSNs encapsulated with permeability glycoprotein (P-gp) siRNA and a polydopamine (PDA) outer layer for FA decoration were designed [91]. Due to the modification of FA, the multifunctional nanodrug-delivery system can actively target tumor cells and achieve the knockout of P-gp in MCF-7/ADR cells.

#### 3.1.5. MSNs Decorated with Polysaccharides

With a molecular weight of 5000–2 × 10^7^ U, hyaluronic acid (HA) is a natural macromolecule acid mucopolysaccharide comprising N-acetylglucosamine and D-glucuronic acid joined by glycosidic bonds [92]. Four HA receptors are distributed on the surface of the cell membrane, namely the CD44 receptor, receptor-mediating HA motility 4 (RHAMM), receptor-mediating HA endocytosis (HARE), and lymphatic endothelial cell HA receptor 1 [93]. According to reports, the CD44 receptor exhibits a difference in expression between normal and tumor cells and is also involved in tumor invasion and cancer cell metastasis. CD44 receptor expression is low in normal tissues and requires activation while it is overexpressed on the surface of several tumor cells and thus can be used as a biomarker for targeting tumors [94]. Through the receptor–ligand interaction, the CD44 receptor precisely binds to HA and its derivatives to achieve targeted delivery.

Ding et al. combined HA and the breast-tumor-cell-penetrating peptide (PEGA-pVEC) as the targeting media in the cascade vector (rmSiO_2_@siRNA@DOX@HA@ peptide NPs, HACT NPs) [95]. To target CD44 overexpressed on the surface of MDA-MB-231 breast cancer cells, rmSiO_2_ was coated with HA, creating a protective shell. The PEGA pVEC peptide, another target ligand for tumor vascular system recognition, is bound to the HA shell by an amino oxime reaction. The assembly of negatively charged peptides was shown by the significant drop in the zeta potential from −5.7 mV to −17.8 mV following the coating of the outermost peptide layer. After an intravenous injection of DDS, NP first accumulates in tumor blood vessels through the peptide’s active targeting recognition of vascular markers and is selectively ingested by CD44-rich cancer cells. Shi and coworkers successfully loaded and delivered an MTH1 inhibitor, TH287, and multidrug resistance protein 1 (MDR1) siRNA with HA-modified MSNs (HA siTMSN) [96]. A cell uptake assay showed that the uptake of rhodamine-labeled HA siTMSN in cancer cells was significant, indicating that it could effectively deliver MDR1 siRNA in the intracellular environment. This firmly establishes HA siTMSN’s dual function in inhibiting MDR1 activity and enhancing the effect of TH287 on tumor-cell killing. Nevertheless, a drawback of using HA as a targeting ligand is that they are high-molecular-weight substances that are rapidly cleared from circulation by liver cells. In an attempt to circumvent this disadvantage, low-molecular-weight HA fragments have been used as targeting moieties. Unfortunately, the affinity of low-molecular-weight HA fragments to the CD44 receptor is lower than that of intact HA, thereby weakening the targeting ability [97].

### 3.2. Facilitation of Endosomal Escape of MSNs

**Figure 3:**
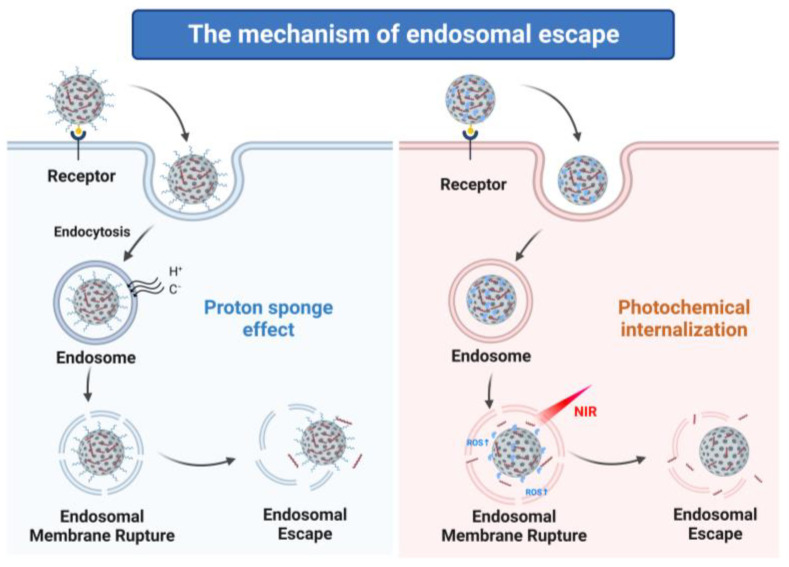
The mechanism of endosomal escape of MSNs.

When siRNA-containing MSNs are internalized by cells through endocytosis, they are transported to late endosomes followed by lysosomes, where they are degraded [98]. Hence, endosomal escape is a prerequisite for siRNA-mediated knockdown to occur. In the study of siRNA delivery systems, how to achieve endosomal escape and release siRNA into the cytoplasm is a crucial factor to consider. The mechanism of endosomal escape mostly includes the proton sponge effect, interaction with the endosomal membrane, and photochemical internalization (Figure 3).

#### 3.2.1. Proton Sponge Effect

According to the proton sponge hypothesis, endosomes inflate due to osmotic pressure and rupture as a result of the buffering power of polycations, releasing their contents into the cytoplasm [99]. Some peptide polymers, such as PEI or PAMAM, usually use the proton sponge effect to achieve the effect of endosome escape [100]. These polymers contain a large number of amino acids, mainly primary and secondary amines, to protonate the endosome during acidification, so as to obtain the pH buffer capacity and inhibit the decline in the environmental pH and enable cells to persist in pumping protons into the endosome to maintain the required pH, which then causes the inward flow of chloride ions and water molecules, leading to the increase in osmotic pressure in the endosome, which then breaks, and the content is released. Parnian and coworkers designed an MSN-based siRNA delivery system by using PEI on the pores’ outer surface and guanidinium ionic liquid (GuIL) groups on the inner surface [101]. It has been demonstrated that PEI and GuIL’s proton sponge effect encourages the endosomal escape of nanoparticles, which is dependent on increased H+ concentrations in endosomes during hydrolysis.

#### 3.2.2. Photochemical Internalization

Photochemical internalization (PCI) is triggered by light to promote drug transport from endosomes to the cytoplasm. The photosensitizer bound to the cytoplasmic membrane enters the cell through the endocytosis pathway and then locates the membrane of the endocytosis vesicles. Under light, the photosensitizer is activated, oxygen-free radicals (ROS) are induced to generate oxygen, the membrane structure of the endosome is destroyed, and drugs are released into the cytoplasm [102]. Zhang and coworkers designed a photo-tearable tape-wrapped nanocapsule by using upconversion nanoparticles (UCNPs) coated with a mesoporous silica layer for the loading of photosensitizer hypocrellin A and siRNA against PLK1 [103]. The UCNPs emit blue emissions in response to exposure to 980 nm of NIR light, which activates HA and causes it to create ROS. ROS make it easier for loaded cargos to escape from endosomes, which increases the efficacy of gene silencing and reduces tumor development in vivo and in vitro.

### 3.3. Promotion of siRNA Dissociate from the MSNs

When delivering siRNA, the main challenges are dissociating siRNA from the carrier to facilitate inclusion into RISC following cellular binding and exiting the endosomes via receptor-mediated endocytosis. Despite continuing efforts to suppress off-target effects and improve the gene-silencing efficiency, understanding the release of siRNA from vectors still possesses vital importance [104].

#### 3.3.1. Chemical Bond Breaking

Through sensitive groups (like acetal bonds, hydrazone bonds, and imine bonds) by cleavage under different conditions, the carrier and the drug are separated, and the release and uptake of the drug are accelerated. The disulfide bond possesses distinct chemical properties [105]. It can be broken down into thiols when exposed to reducing substances that contain sulfhydryl functional groups, such as glutathione (GSH). The responsiveness of the bond to different concentrations of GSH varies significantly, allowing for controlled release in the vector. Lu and coworkers described and designed a redox-responsive MSN with enlarged pores (denoted as MSN-siRNA/CrPEI) by immobilizing PEI as a cap for redox-responsive intracellular RNA delivery via intermediate linkers of disulfide attached to the MSNs [106]. After MSNs were loaded with siRNAs in the mesopores, a positively charged polymer PEI was utilized as a supramolecular cap on the surface of MSNs via disulfide linkages induced by dithiobis(succinimidyl propionate) and disulfide-bond crosslinked PEI to retain cargo within the MSNs. The closed capping PEI on the MSN surface was swiftly removed after the siRNA-loaded MSNs were internalized by the tumor cell because the disulfide bond was broken in the presence of the GSH released by the tumor cell. In the presence of dithiothreitol, a small reductive molecule used to simulate the effect of GSH, a burst release of siRNA due to the cleavage of the disulfide bond was observed. At a pH of 7.4, the cumulative drug release percentage reached up to 90%, whereas no siRNAs could be found within 120 h without dithiothreitol.

#### 3.3.2. The Change in Solubility

Through the change in solubility of the dissociable groups (such as the amino and carboxyl groups) on the polymer, the stability of the carrier system reduces while drug release is promoted. A surface carboxylated MSN formed an in situ bond with a zeolite imidazole framework-8 (ZIF-8) membrane with a thickness of several nanometers and was used to block the pores of the MSN to efficiently load siRNAs for a pH-responsive drug-delivery system [107,108]. The ZIF-8 membrane can effectively load Bcl-2 siRNA via electrostatic interactions, alter the charge of MSN-COOH from negative to positive, and shield siRNAs from nuclease degradation. At a pH of 6.0, siRNA-releasing profiles show a fast siRNA release in the initial 3 h and remain unchanged for 8 h. In contrast, no obvious release of siRNA is observed at a pH of 7.4 for 8 h. The quick reaction and elevated release can be linked to the ZIF-8 film’s quick breakdown under acidic circumstances. MSN-COOH@ZIF-8 NPs enable the rapid cellular uptake of siRNAs, and ultrathin ZIF-8 membranes can be broken down in acidic lysosomes, thereby triggering the intracellular release of siRNAs, which significantly enhances the therapeutic effects of cancer cells including MCF-7/ADR and SKOV-3/ADR cells.

## 4. siRNA-Loaded MSNs for Cancer Treatment

Research on siRNA in the field of antitumor gene therapy has been increasing, making it a major focus in cancer treatment. By targeting specific genes and effectively inhibiting the expression of disease-causing genes (such as oncogenes) and/or regulating the expression of tumor-suppressor genes, it is possible to achieve the prevention and treatment of tumors. siRNA provides an efficient means to silence specific genes, and its recognition of target sequences is highly specific. By chemically synthesizing siRNA targeting different genes and transfecting them into tumor cells, tumor signal transduction can be disrupted and the relevant target genes can be modulated, thereby achieving the goal of blocking tumor occurrence and development [14,109,110,111].

### 4.1. Prevention of Multiple-Drug Resistance

**Figure 4:**
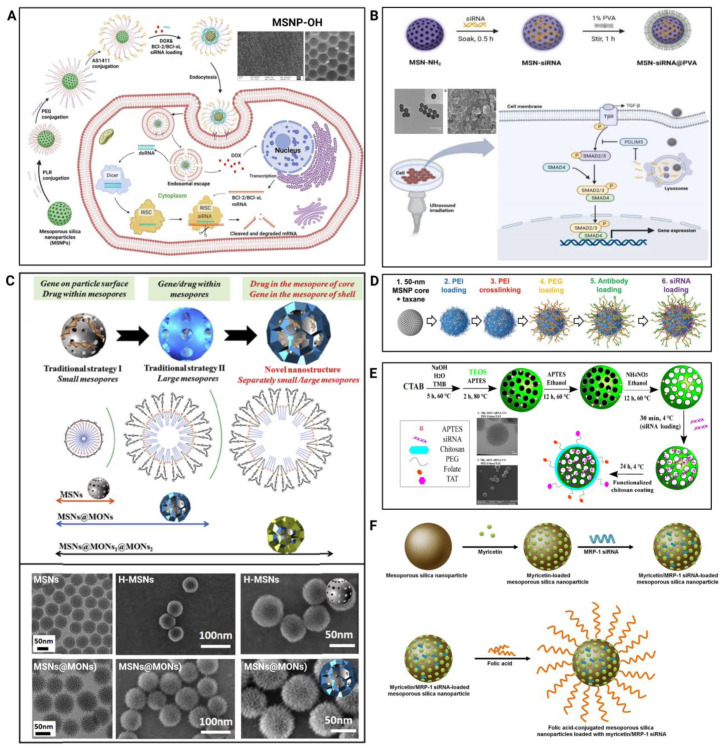
Loaded and tumor-targeted delivery of siRNAs via MSNs for overcoming drug resistance. (**A**) Schematic representation of the synthesis of MSNP-PLR-PEG(OCH3) and overcoming resistance through the BCl-2/BCl-xL pathway; (**B**) schematic illustration of the preparation process and antagonistic drug-resistance strategies of siRNA-MSN@PVA NPs; (**C**) structural evolution of MSNs from small pore-sized MSNs to large pore-sized MSNs, the hierarchical MSNs in present work for gene/drug codelivery for MDR reversing, and the schematic illustration of the formation mechanism of MSNs@MONs; (**D**) schematic of siRNA-MSNs nanoconstruct synthesis; (**E**) schematic illustration of synthesis steps and MSNs functionalization to obtain NH2-MSN, NH2-MSN-siRNA, and NH2-MSN-siRNA-chitosan functionalized with PEG-folate and PEG-TAT; (**F**) the preparation of folic-acid-conjugated mesoporous silica nanoparticles loaded with myricetin and MRP-1 siRNA.

Cancers can frequently develop treatment resistance because of their highly heterogeneous character [112]. Chemotherapy has long been utilized as a first-line treatment for numerous malignancies [113]. The development of tumor heterogeneity and treatment resistance, however, reduces the efficacy of several routinely used cancer therapies. Changes in drug influx/efflux, the augmentation of the DNA repair ability, transformations in drug metabolism (to achieve detoxification), drug target mutation, and the activation of parallel signaling pathways are all examples of MDR [110,114,115]. The combination of two drugs that can block many survival pathways in tumor cells simultaneously is particularly useful for tackling drug resistance and improving the antitumor ability [116,117]. By restoring tumor-suppressor genes and introducing apoptotic genes, the combination of RNAi and chemotherapy drugs can augment chemotherapy in several ways. The codelivery of siRNAs and anticancer drugs results in a synergistic impact; once the siRNA silences the targeted gene, drug efflux pumps reduce, thus allowing drug diffusion into cells across cell membranes [118,119,120,121,122] (Figure 4). Hence, the apoptotic process is accelerated, resulting in enhanced chemotherapeutic effects.

Resistance to antineoplastic drugs mostly comprises pump and nonpump drug resistance. Pump resistance is caused by the ATP-binding cassette (ABC) transporters, such as P-gp, MDR-related protein, and ABC subfamily G member 2 (ABCG2), which carry drugs from cancer cells to the plasma membrane [123]. To overcome resistance to antineoplastic drugs, a stimuli-responsive delivery system (HPMSN) with tumor-targeting capabilities to codeliver DOX and GCN5 siRNA (siGCN5) was developed [124]. This system efficiently transported DOX and siGCN5 into drug-resistant cancer cells, and the siGCN5 released later downregulated P-gp expression and suppressed the DOX efflux. The HPMSN jointly delivered by DOX/siGCN5 effectively inhibited tumor growth by 77% and reduced DOX systemic toxicity in the MDR breast tumor model (MCF7/ADR). In another study, through loaded MSNs, antineoplastic drugs and anti-ABCG2 siRNAs were successfully delivered to CD133+ cancer cells [125]. The downregulation of ABCG2 considerably improved the efficacy of chemotherapeutic drugs in inducing the apoptosis of laryngeal cancer cells, along with the efficacy of therapeutic agents in inducing apoptosis in mice models of laryngeal cancer.

### 4.2. Induction of Cancer Apoptosis

**Figure 5:**
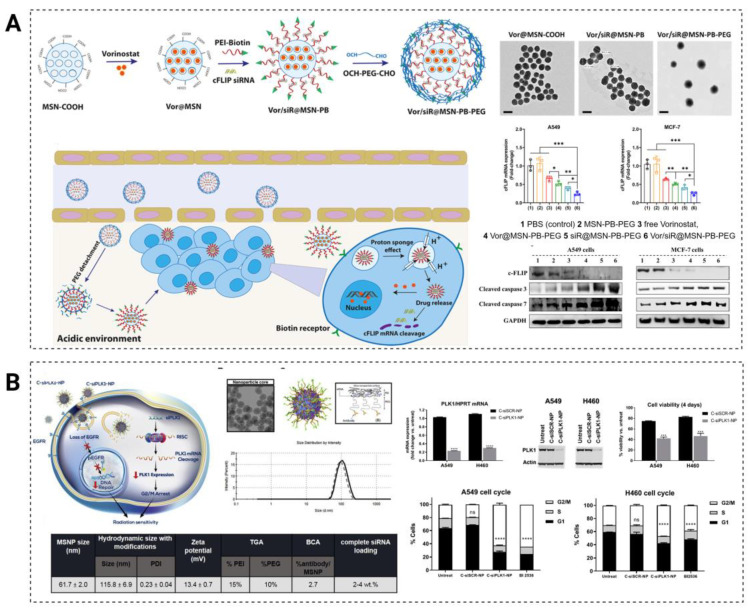
Promotion of apoptosis in tumor cells by MSNs-loaded siRNAs. (**A**) Scheme of the preparation process of Vorinostat and cFLIP siRNA-coloaded mesoporous silica nanoparticles with pH-ultrasensitive valves (Vor/siR@MSN-PB-PEG) for suppression of cFLIP in cancer cells, Vor/siR@MSN-PB-PEG-inhibited cFLIP expression, and induced apoptosis caspase pathways in cancer cells; (**B**) EGFR-targeted (cetuximab) mesoporous silica nanoparticle (NP) platform for PLK1 siRNA (siPLK1) delivery or C-siPLK1-NP and proapoptotic effects of C-siPLK1-NP treatment on NSCLC (A549, H460) cell lines. * *p* < 0.05, ** *p* < 0.01, *** *p* < 0.001, **** *p* < 0.0001, ns = not statistically significant.

In the development of tumors, certain genes such as oncogenes and tumor-suppressor genes play a crucial role in controlling programmed cell death, also known as cellular apoptosis. When these genes are not properly regulated, they can lead to uncontrolled cell growth and prevent the normal apoptotic process, ultimately resulting in cellular malignancy and unlimited proliferation [126,127,128]. As research on apoptosis deepens and key apoptotic molecules are discovered, “promoting tumor cell apoptosis” has become an important strategy in anticancer therapy [129,130] (Figure 5).

Bcl-2, an antiapoptotic gene closely associated with malignant tumors, can impede the apoptosis of tumor cells induced by chemotherapy drugs and is closely linked to cancer development and drug resistance [131]. By utilizing RNAi technology to construct corresponding siRNA vectors targeting different hepatocellular carcinoma genes, the expression of cancer-related genes can be inhibited, leading to the induction of cancer cell apoptosis. This approach selectively blocks the transcription products of oncogenes without affecting the expression of other genes [132]. Choi and coworkers loaded chloroquine (CQ) into the pores of calcium-doped MSNs and subsequently loaded Bcl-2 siRNA in an amination-free manner [133]. This delivery system increased the sensitivity of SKOV3 human ovarian cancer cells to CQ due to the siRNA-induced downregulation of Bcl-2 expression and showed an enhanced therapeutic efficacy. Besides Bcl-2, other proteins are closely associated with apoptosis in tumor cells. Alpha-fetoprotein (AFP) is a specific protein that produces multiple effects on cell differentiation, proliferation, and tumor occurrence. The stable expression of siRNA-AFP has been established in vitro, and by silencing the AFP gene and upregulating Caspase-3 expression, significant apoptosis induction has been observed in cancer cells [134]. By designing MSN vectors for the appropriate siRNAs, it is feasible to effectively manage the expression of genes associated with cancer and trigger apoptosis in cancer cells.

### 4.3. Inhibition of Angiogenesis

The growth of malignant solid tumors is dependent on angiogenesis, which occurs after the tumor breaches the basement membrane. This process enables the tumor to acquire a continuous supply of nutrients and oxygen, facilitating its growth and expansion. Neovascularization is therefore an important factor to consider in the treatment of tumors. The formation of new blood vessels is closely linked to tumor growth, proliferation, and metastasis. By preventing the development of tumor neovasculature and cutting off the tumor’s nutrient supply, we can more effectively inhibit tumor invasion, recurrence, and metastasis [135]. The amplification of growth factor and receptor expression, such as Epithelial Growth Factor (EGF), Insulin-like Growth Factor (IGF), Vascular Endothelial Growth Factor (VEGF), and their receptors’ expression, is closely associated with cell overgrowth and angiogenesis [136,137]. Due to angiogenesis’ pivotal function in the growth and propagation of tumors, VEGF has become a hot topic in antitumor angiogenesis research. Using VEGF siRNA to downregulate VEGF is a promising cancer treatment that inhibits tumor angiogenesis and metastasis [8]. Chen et al. successfully inhibited the expression of the VEGF gene at the site of ovarian tumors by constructing M-MSN_siRNA@PEI-PEG-KALA loaded with VEGF siRNA [138]. Due to the significant reduction in angiogenesis, there was a noticeable suppression of in situ ovarian tumor growth without any systemic toxicity. Sun et al. designed an oxidoreductive-responsive MSN delivery system (MSN-siRNA/CrPEl) that released VEGF siRNA from the cytoplasmic MSN in response to high levels of GSH present at the tumor site, thereby exerting its gene-silencing effect [106]. VEGF siRNA, through gene silencing, reduction in the tumor interstitial fluid pressure, inhibition of CD31, and suppression of angiogenesis had been applied to KB tumor-bearing mice by using this system. It had been demonstrated to have a significant anticancer effect.

### 4.4. Effective Activation of Immunity

**Figure 6:**
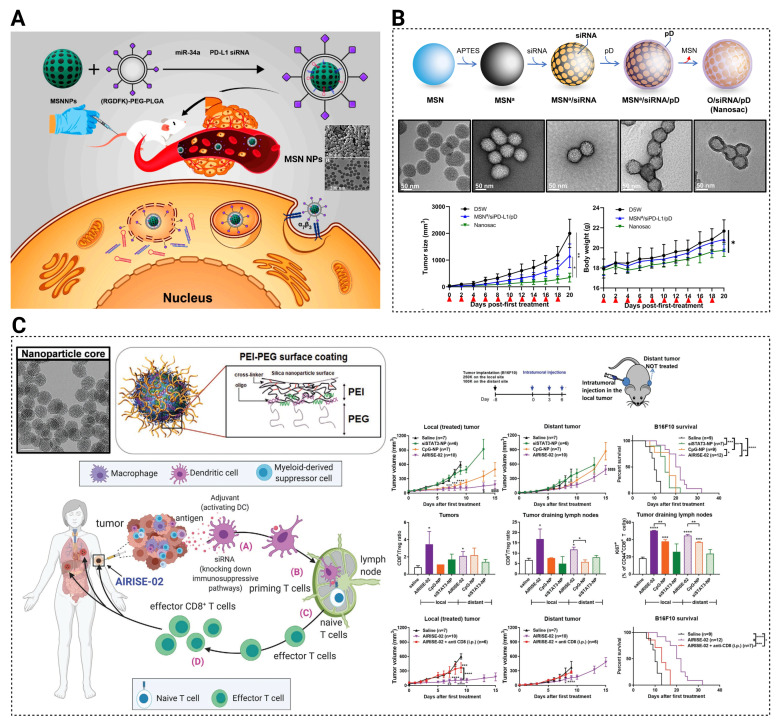
Effective activation of immunity by MSNs loaded with siRNAs. (**A**) Preparation and intracellular behavior of c(RGDfK)-MSN; (**B**) schematic and TEM images of O/siRNA/pD (Nanosac) preparation and antitumor activity of Nanosac in Balb/c mice bearing CT26 tumors; (**C**) schematic and TEM images of siSTAT3-CpG-NP and its TEM electron micrographs. Effectiveness of siSTAT3–CpG–NP in inducing in situ tumor vaccination in mice bearing bilateral syngeneic melanoma tumors. * *p* < 0.05, ** *p* < 0.01, *** *p* < 0.001, **** *p* < 0.0001.

Immunotherapy approaches have gained attention in the field of cancer treatment as they have the potential to eradicate cancer cells and metastatic tumors by recruiting the host immune system. Unfortunately, not all therapeutic targets can be effectively treated with antibodies or small molecule inhibitors due to the complex mechanisms of immune evasion by tumor cells. RNAi technology, which can target different molecules, has been used to silence specific targets in tumor cells and noncancerous host cells, enhancing the immune response against tumors [139]. Some cancer cells produce molecules that help them evade the immune system (Figure 6). These molecules can be targeted for gene silencing by using siRNA. The signal transducer and activator of transcription 3 (STAT3) plays a pivotal role in immune suppression in tumors. Inhibiting STAT3 in cancer cells enhances immunogenic cell death and increases the production of interferon-responsive chemokines that facilitate immune cell infiltration [140]. Furthermore, STAT3 also mediates immune inhibitory functions in various tumor-associated immune cells, such as eliminating STAT3 in dendritic cells, and can improve antigen presentation activity and enhance adaptive antitumor immune responses [141]. Researchers have devised a tumor vaccine named AIRISE-02 that utilizes MSNs as carriers to codeliver CpG and siSTAT3 [142]. This innovative vaccine aims to augment immune responses within the tumor microenvironment and counteract its inhibitory effects. Remarkably, AIRISE-02 has exhibited a remarkable therapeutic efficacy in models of melanoma, breast cancer, and colon cancer. The use of immune checkpoint inhibitors has greatly advanced tumor immunotherapy, but their effectiveness in treating many cancers is still limited. Programmed cell death protein 1 (PD1) is an immune checkpoint inhibitor that suppresses T lymphocyte function by binding to programmed cell death protein ligand-1 (PDL1), resulting in a reduced self-immune response [143]. Researchers have developed a siRNA carrier called “Nanosac” by coating MSNs with polydopamine and then removing the sacrificial MSN core [62]. Nanosac acts as a targeted siRNA carrier for PD-L1, successfully hindering the growth of CT26 tumors by inducing immune checkpoint blockades.

### 4.5. Combination with Other Therapies

**Figure 7:**
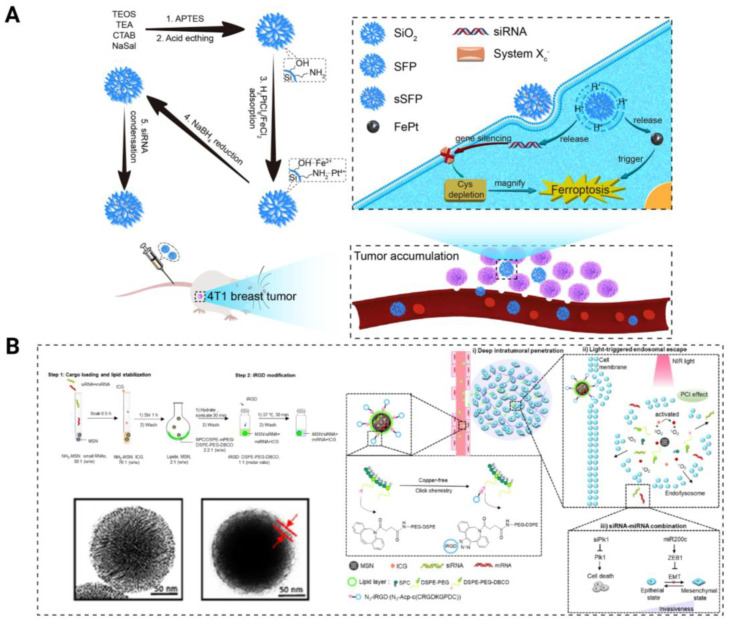
Combining multiple therapies against tumors through MSN delivery of siRNAs. (**A**) Preparation of FePt nanoparticles and siRNA coloaded mesoporous silica nanoplatform sSFP and enhanced ferroptosis process of tumor cells by Cys depletion; (**B**) schematic illustration of light-triggered RNA delivery by tumor-penetrating iMSNs for siPlk1/miR-200c combination therapy.

Ferroptosis is a new form of programmed cell death that involves iron-dependent lipid peroxidation and is linked to various illnesses, such as cancer [144]. Encouraging ferroptosis in cancer cells could be an effective cancer-treatment method. Consequently, ferroptosis-inducing drugs are gaining more recognition in cancer treatment. Li and coworkers created a platform called siRNA@SFP (sSFP) by using mesoporous silica nanoparticles [70]. This platform enhances ferroptosis by reducing the levels of cysteine (Figure 7A). They synthesized FePt nanoparticles within the pore channels of dendritic mesoporous silica nanoparticles and efficiently loaded siRNA targeting xCT onto them through electrostatic interactions. The iron death-inducer FePt within sSFP nanoparticles elicits ferroptosis in breast tumor cells. By interfering with the expression of xCT and inhibiting cysteine uptake, siRNA effectively depletes intracellular cysteine levels. This depletion disrupts the redox system and magnifies the cytotoxic impact of ferroptosis on tumor cells.

Photothermal therapy (PTT), an emerging cancer-treatment method, uses photothermal agents to convert absorbed NIR light into thermal energy to achieve the photothermal ablation of cells [145]. By injecting materials with a high photothermal conversion efficiency into the human body and using targeted recognition technology to accumulate them near tumor tissues, it is possible to convert light energy into heat energy. This approach is designed to selectively and precisely eliminate cancer cells with minimal invasiveness when exposed to external light irradiation. The FDA has approved the clinical use of the photosensitizer indocyanine green (ICG) with outstanding biocompatibility. It is suitable for PTT/PDT and can efficiently absorb light energy to produce singlet oxygen or heat energy. ICG loaded into MSNs for local ROS production improves cytosolic siRNA delivery. iMSNs (loaded ICGs) with light-triggered endosomal escape and tumor-penetration capacities substantially increase the RNA delivery efficacy [70]. Researchers have devised a versatile MSN delivery system capable of codelivering siPlk1 and miRNA (miR-200c), thereby integrating photothermal therapy with RNA interference (Figure 7B). By downregulating the expression of Plk1, a critical mitotic factor in the tumor cell cycle, siPlk1 inhibits tumor progression. The combined delivery of therapeutic RNAs demonstrates synergistic cytotoxic activity in metastatic breast cancer and reduces metastasis following transient in situ light exposure.

## 5. Conclusions and Outlook

RNAi therapies work by directly introducing genes—either through regulation or replacement—into cancer cells or the tissues around a tumor. However, the vulnerability of degradation by serum nucleases, the difficulty of crossing physiological barriers, the potential off-target effect, and the possibility of activating the innate immune system has limited clinical applicability. As relatively well-studied and widely used nanomaterials, MSNs possess characteristics such as different pore sizes, good biocompatibility, and various targeting strategies, making them a suitable carrier for siRNA. The incorporation of siRNAs onto nanometer-sized MSNs has clearly advanced their biological behavior. siRNA-loaded MSNs have greatly overcome the aforementioned limitations and provided a new strategy to tackle cancer. To realize clinical functionalization, a therapeutic combination was promoted recently to extend synergistic antitumor effects by using RNAi and other adjuvants, which can induce ROS or heat species to achieve the ablation of cells. Additionally, through various forms of surface functionalization and the creation of core/shell nanomaterials, therapeutic techniques using MSN-based RNAi are being successfully integrated with various imaging modalities. We anticipate that these functional MSN systems have an excellent application potential for RNAi applications, such as achieving precise treatment and the integration of cancer diagnosis and treatment. In conclusion, the development of multifunctionally modified MSNs or their combination with other functional biomolecules has led to the pursuit of smart nanomedicine based on MSNs, and the testing of these systems in depth in vitro and in vivo has grown to be one of the most significant study fields in recent years. Future developments will see siRNA-loaded MSNs used in a wider range of drug-delivery procedures, therapy, and therapeutic evaluation.

## Data Availability

Not applicable.

## Abbreviations

ABC, ATP-binding cassette; ABCG2, ABC subfamily G member 2; AAVs, adeno-associated viruses; APTES, triethoxysilane; CD47, cluster of differentiation 47; CQ, chloroquine; DOX, doxorubicin; EPR, enhanced permeability and retention; FDA, the Food and Drug Administration; FR, folate receptor; G2 PAMAMs, second-generation polyamidoamines; GSH, glutathione; GuIL, guanidinium ionic liquid; HA, hyaluronic acid; HARE, receptor-mediating HA endocytosis; HER2, human epidermal growth factor receptor 2; ICG, indocyanine green; MDR1, multidrug resistance protein 1; MSNs, mesoporous silica nanoparticles; NPs, nanoparticles; PCI, photochemical internalization; PDA, polydopamine; PD-L1, programmed cell death ligand-1; PDT, photodynamic therapy; PEI, polyethyleneimine; PGA, pteroylglutamic acid; P-gp, permeability glycoprotein; PLK1, polo-like kinase 1; PLL, poly-l-lysine; PTK, protein tyrosine kinase; PTT, photothermal therapy; RGD, Arg-Gly-Asp; RHAMM, receptor-mediating HA motility; RISC, RNA-induced silencing complex; RNAi, RNA interference; siRNA, small interfering RNA; ROS, oxygen-free radicals; STAT3, signal transducer and activator of transcription 3; TGF-β, transforming growth factor beta; VEGF, Vascular Endothelial Growth Factor; ZIF-8, zeolite imidazole framework-8.

## References

[1] Adams D., Gonzalez-Duarte A., O’Riordan W.D., Yang C.C., Ueda M., Kristen A.V., Tournev I., Schmidt H.H., Coelho T., Berk J.L. (2018). Patisiran, an RNAi Therapeutic, for Hereditary Transthyretin Amyloidosis. N. Engl. J. Med..

[2] Paunovska K., Loughrey D., Dahlman J.E. (2022). Drug delivery systems for RNA therapeutics. Nat. Rev. Genet..

[3] Kara G., Calin G.A., Ozpolat B. (2022). RNAi-based therapeutics and tumor targeted delivery in cancer. Adv. Drug Deliv. Rev..

[4] Won J.E., Byeon Y., Wi T.I., Lee C.M., Lee J.H., Kang T.H., Lee J.-W., Lee Y., Park Y.-M., Han H.D. (2022). Immune checkpoint silencing using RNAi-incorporated nanoparticles enhances antitumor immunity and therapeutic efficacy compared with antibody-based approaches. J. Immunother. Cancer.

[5] Huang K.-W., Hsu F.-F., Qiu J.T., Chern G.-J., Lee Y.-A., Chang C.-C., Huang Y.-T., Sung Y.-C., Chiang C.-C., Huang R.-L. (2020). Highly efficient and tumor-selective nanoparticles for dual-targeted immunogene therapy against cancer. Sci. Adv..

[6] Li Y., Ding J., Xu X., Shi R., Saw P.E., Wang J., Chung S., Li W., Aljaeid B.M., Lee R.J. (2020). Dual Hypoxia-Targeting RNAi Nanomedicine for Precision Cancer Therapy. Nano Lett..

[7] Yang Y., Meng Y., Ye J., Xia X., Wang H., Li L., Dong W., Jin D., Liu Y. (2018). Sequential delivery of VEGF siRNA and paclitaxel for PVN destruction, anti-angiogenesis, and tumor cell apoptosis procedurally via a multi-functional polymer micelle. J. Control. Release.

[8] Jin M., Zeng B., Liu Y., Jin L., Hou Y., Liu C., Liu W., Wu H., Chen L., Gao Z. (2022). Co-Delivery of Repurposing Itraconazole and VEGF siRNA by Composite Nanoparticulate System for Collaborative Anti-Angiogenesis and Anti-Tumor Efficacy against Breast Cancer. Pharmaceutics.

[9] Gregory J.V., Kadiyala P., Doherty R., Cadena M., Habeel S., Ruoslahti E., Lowenstein P.R., Castro M.G., Lahann J. (2020). Systemic brain tumor delivery of synthetic protein nanoparticles for glioblastoma therapy. Nat. Commun..

[10] Lin Y.-X., Wang Y., Blake S., Yu M., Mei L., Wang H., Shi J. (2020). RNA Nanotechnology-Mediated Cancer Immunotherapy. Theranostics.

[11] Zhang Q., Hossain D.M.S., Nechaev S., Kozlowska A., Zhang W., Liu Y., Kowolik C.M., Swiderski P., Rossi J.J., Forman S. (2013). TLR9-mediated siRNA delivery for targeting of normal and malignant human hematopoietic cells in vivo. Blood.

[12] Fan X., Gu L., Lv S., Zhang M., Zhuang L., Zhang Y., Chen P. (2022). Suppression of the transforming growth factor-β signaling pathway produces a synergistic effect of combination therapy with programmed death receptor 1 blockade and radiofrequency ablation against hepatic carcinoma in mice. Bioengineered.

[13] Yonezawa S., Koide H., Asai T. (2020). Recent advances in siRNA delivery mediated by lipid-based nanoparticles. Adv. Drug Deliv. Rev..

[14] Zhang M., Huang Y. (2022). siRNA modification and delivery for drug development. Trends Mol. Med..

[15] Pupo A., Fernández A., Low S.H., François A., Suárez-Amarán L., Samulski R.J. (2022). AAV vectors: The Rubik’s cube of human gene therapy. Mol. Ther..

[16] Aronson S.J., Veron P., Collaud F., Hubert A., Delahais V., Honnet G., de Knegt R.J., Junge N., Baumann U., Di Giorgio A. (2019). Prevalence and Relevance of Pre-Existing Anti-Adeno-Associated Virus Immunity in the Context of Gene Therapy for Crigler–Najjar Syndrome. Hum. Gene Ther..

[17] Bryson T.E., Anglin C.M., Bridges P.H., Cottle R.N. (2017). Nuclease-Mediated Gene Therapies for Inherited Metabolic Diseases of the Liver. Yale J. Biol. Med..

[18] Nguyen G.N., Everett J.K., Kafle S., Roche A.M., Raymond H.E., Leiby J., Wood C., Assenmacher C.-A., Merricks E.P., Long C.T. (2021). A long-term study of AAV gene therapy in dogs with hemophilia A identifies clonal expansions of transduced liver cells. Nat. Biotechnol..

[19] Challis R.C., Kumar S.R., Chan K.Y., Challis C., Beadle K., Jang M.J., Kim H.M., Rajendran P.S., Tompkins J.D., Shivkumar K. (2019). Systemic AAV vectors for widespread and targeted gene delivery in rodents. Nat. Protoc..

[20] Yan Y., Liu X.-Y., Lu A., Wang X.-Y., Jiang L.-X., Wang J.-C. (2022). Non-viral vectors for RNA delivery. J. Control. Release.

[21] Arshad R., Fatima I., Sargazi S., Rahdar A., Karamzadeh-Jahromi M., Pandey S., Díez-Pascual A.M., Bilal M. (2021). Novel Perspectives towards RNA-Based Nano-Theranostic Approaches for Cancer Management. Nanomaterials.

[22] Wang M., Zhao J., Jiang H., Wang X. (2022). Tumor-targeted nano-delivery system of therapeutic RNA. Mater. Horizons.

[23] Zhao W., Hou X., Vick O.G., Dong Y. (2019). RNA delivery biomaterials for the treatment of genetic and rare diseases. Biomaterials.

[24] Fan H., Sun Q., Dukenbayev K., Benassi E., Manarbek L., Nurkesh A.A., Khamijan M., Mu C., Li G., Razbekova M. (2022). Carbon nanoparticles induce DNA repair and PARP inhibitor resistance associated with nanozyme activity in cancer cells. Cancer Nanotechnol..

[25] Hu H., Yang C., Zhang F., Li M., Tu Z., Mu L., Dawulieti J., Lao Y., Xiao Z., Yan H. (2021). A Versatile and Robust Platform for the Scalable Manufacture of Biomimetic Nanovaccines. Adv. Sci..

[26] Barkat A., Beg S., Panda S.K., Alharbi K.S., Rahman M., Ahmed F.J. (2021). Functionalized mesoporous silica nanoparticles in anticancer therapeutics. Semin. Cancer Biol..

[27] Hanafi-Bojd M.Y., Ansari L., Malaekeh-Nikouei B. (2016). Codelivery of anticancer drugs and siRNA by mesoporous silica nanoparticles. Ther Deliv..

[28] Khaliq N.U., Lee J., Kim J., Kim Y., Yu S., Kim J., Kim S., Sung D., Kim H. (2023). Mesoporous Silica Nanoparticles as a Gene Delivery Platform for Cancer Therapy. Pharmaceutics.

[29] Niu Y., Yu M., Hartono S.B., Yang J., Xu H., Zhang H., Zhang J., Zou J., Dexter A., Gu W. (2013). Nanoparticles mimicking viral surface topography for enhanced cellular delivery. Adv. Mater..

[30] Isa E.D.M., Ahmad H., Rahman M.B.A., Gill M.R. (2021). Progress in Mesoporous Silica Nanoparticles as Drug Delivery Agents for Cancer Treatment. Pharmaceutics.

[31] Dawulieti J., Sun M., Zhao Y., Shao D., Yan H., Lao Y.-H., Hu H., Cui L., Lv X., Liu F. (2020). Treatment of severe sepsis with nanoparticulate cell-free DNA scavengers. Sci. Adv..

[32] Li T., Shi S., Goel S., Shen X., Xie X., Chen Z., Zhang H., Li S., Qin X., Yang H. (2019). Recent advancements in mesoporous silica nanoparticles towards therapeutic applications for cancer. Acta Biomater..

[33] Kim J.-S., Lee S.K., Doh H., Kim M.Y., Kim D.K. (2021). Real-Time Tracking of Highly Luminescent Mesoporous Silica Particles Modified with Europium β-Diketone Chelates in Living Cells. Nanomaterials.

[34] Braun K., Stürzel C.M., Biskupek J., Kaiser U., Kirchhoff F., Lindén M. (2018). Comparison of different cytotoxicity assays for in vitro evaluation of mesoporous silica nanoparticles. Toxicol. In Vitro.

[35] Cao H., Yang Y., Chen X., Shao Z. (2016). Intelligent Janus nanoparticles for intracellular real-time monitoring of dual drug release. Nanoscale.

[36] Živojević K., Mladenović M., Djisalov M., Mundzic M., Ruiz-Hernandez E., Gadjanski I., Knežević N.Ž. (2021). Advanced mesoporous silica nanocarriers in cancer theranostics and gene editing applications. J. Control. Release.

[37] Kankala R.K., Han Y., Na J., Lee C., Sun Z., Wang S., Kimura T., Ok Y.S., Yamauchi Y., Chen A. (2020). Nanoarchitectured Structure and Surface Biofunctionality of Mesoporous Silica Nanoparticles. Adv. Mater..

[38] Gong J., Wang H., Lao Y., Hu H., Vatan N., Guo J., Ho T., Huang D., Li M., Shao D. (2020). A Versatile Nonviral Delivery System for Multiplex Gene-Editing in the Liver. Adv. Mater..

[39] Li Q., Hao X., Zaidi S.S.A., Guo J., Ren X., Shi C., Zhang W., Feng Y. (2018). Oligohistidine and targeting peptide functionalized TAT-NLS for enhancing cellular uptake and promoting angiogenesis in vivo. J. Nanobiotechnol..

[40] Sun Y., Ma X., Jing X., Hu H. (2021). PAMAM-Functionalized Cellulose Nanocrystals with Needle-Like Morphology for Effective Cancer Treatment. Nanomaterials.

[41] Chan M., Huang W., Wang J., Liu R., Hsiao M. (2020). Next-Generation Cancer-Specific Hybrid Theranostic Nanomaterials: MAGE-A3 NIR Persistent Luminescence Nanoparticles Conjugated to Afatinib for In Situ Suppression of Lung Adenocarcinoma Growth and Metastasis. Adv. Sci..

[42] Yao Q., Liu Y., Selvaratnam B., Koodali R.T., Sun H. (2018). Mesoporous silicate nanoparticles/3D nanofibrous scaffold-mediated dual-drug delivery for bone tissue engineering. J. Control. Release.

[43] Meka A.K., Niu Y., Karmakar S., Hartono S.B., Zhang J., Lin C.X.C., Zhang H., Whittaker A., Jack K., Yu M. (2016). Facile Synthesis of Large-Pore Bicontinuous Cubic Mesoporous Silica Nanoparticles for Intracellular Gene Delivery. ChemNanoMat.

[44] Mora-Raimundo P., Lozano D., Manzano M., Vallet-Regí M. (2019). Nanoparticles to Knockdown Osteoporosis-Related Gene and Promote Osteogenic Marker Expression for Osteoporosis Treatment. ACS Nano.

[45] Dilnawaz F., Sahoo S.K. (2018). Augmented Anticancer Efficacy by si-RNA Complexed Drug-Loaded Mesoporous Silica Nanoparticles in Lung Cancer Therapy. ACS Appl. Nano Mater..

[46] Kamegawa R., Naito M., Miyata K. (2018). Functionalization of silica nanoparticles for nucleic acid delivery. Nano Res..

[47] Badihi R., Mahmoudi A., Sazegar M.R., Nazari K. (2022). A study on co-modification of MSNs with some transition metals and polyethyleneimine (PEI) as a versatile strategy for efficient delivery of short oligonucleotides. Chem. Pap..

[48] Chen A.M., Zhang M., Wei D., Stueber D., Taratula O., Minko T., He H. (2009). Co-delivery of doxorubicin and Bcl-2 siRNA by mesoporous silica nanoparticles enhances the efficacy of chemotherapy in multidrug-resistant cancer cells. Small.

[49] Chang L., Yan H., Chang J., Gautrot J.E. (2021). Cationic polymer brush-coated bioglass nanoparticles for the design of bioresorbable RNA delivery vectors. Eur. Polym. J..

[50] El-Kharrag R., Halim S.S.A., Amin A., Greish Y.E. (2019). Synthesis and characterization of chitosan-coated magnetite nanoparticles using a modified wet method for drug delivery applications. Int. J. Polym. Mater. Polym. Biomater..

[51] Na H.K., Kim M.H., Park K., Ryoo S.R., Lee K.E., Jeon H., Ryoo R., Hyeon C., Min D.H. (2012). Efficient functional delivery of siRNA using mesoporous silica nanoparticles with ultralarge pores. Small.

[52] Shakeran Z., Varshosaz J., Keyhanfar M., Mohammad-Beigi H., Rahimi K., Sutherland D.S. (2022). Co-delivery of STAT3 siRNA and methotrexate in breast cancer cells. Artif. Cells Nanomed. Biotechnol..

[53] Cha W., Fan R., Miao Y., Zhou Y., Qin C., Shan X., Wan X., Li J. (2017). Mesoporous Silica Nanoparticles as Carriers for Intracellular Delivery of Nucleic Acids and Subsequent Therapeutic Applications. Molecules.

[54] Ashley C.E., Carnes E.C., Epler K.E., Padilla D.P., Phillips G.K., Castillo R.E., Wilkinson D.C., Wilkinson B.S., Burgard C.A., Kalinich R.M. (2012). Delivery of small interfering RNA by peptide-targeted mesoporous silica nanoparticle-supported lipid bilayers. ACS Nano.

[55] Li X., Chen Y., Wang M., Ma Y., Xia W., Gu H. (2013). A mesoporous silica nanoparticle--PEI--fusogenic peptide system for siRNA delivery in cancer therapy. Biomaterials.

[56] Lin D., Cheng Q., Jiang Q., Huang Y., Yang Z., Han S., Zhao Y., Guo S., Liang Z., Dong A. (2013). Intracellular cleavable poly(2-dimethylaminoethyl methacrylate) functionalized mesoporous silica nanoparticles for efficient siRNA delivery in vitro and in vivo. Nanoscale.

[57] Hartono S.B., Yu M., Gu W., Yang J., Strounina E., Wang X., Qiao S., Yu C. (2014). Synthesis of multi-functional large pore mesoporous silica nanoparticles as gene carriers. Nanotechnology.

[58] Li Y., Hei M., Xu Y., Qian X., Zhu W. (2016). Ammonium salt modified mesoporous silica nanoparticles for dual intracellular-responsive gene delivery. Int. J. Pharm..

[59] Xiong L., Bi J., Tang Y., Qiao S. (2016). Magnetic Core–Shell Silica Nanoparticles with Large Radial Mesopores for siRNA Delivery. Small.

[60] Lio D.C.S., Liu C., Oo M.M.S., Wiraja C., Teo M.H.Y., Zheng M., Chew S.W.T., Wang X., Xu C. (2019). Transdermal delivery of small interfering RNAs with topically applied mesoporous silica nanoparticles for facile skin cancer treatment. Nanoscale.

[61] Möller K., Müller K., Engelke H., Bräuchle C., Wagner E., Bein T. (2016). Highly efficient siRNA delivery from core–shell mesoporous silica nanoparticles with multifunctional polymer caps. Nanoscale.

[62] Kim H., Yuk S.A., Dieterly A.M., Kwon S., Park J., Meng F., Gadalla H.H., Cadena M.J., Lyle L.T., Yeo Y. (2021). Nanosac, a Noncationic and Soft Polyphenol Nanocapsule, Enables Systemic Delivery of siRNA to Solid Tumors. ACS Nano.

[63] Shi P., Li M., Song C., Qi H., Ba L., Cao Y., Zhang M., Xie Y., Ren J., Wu J. (2022). Neutrophil-like cell membrane-coated siRNA of lncRNA AABR07017145.1 therapy for cardiac hypertrophy via inhibiting ferroptosis of CMECs. Mol. Ther. Nucleic Acids.

[64] Hao N., Jayawardana K.W., Chen X., Yan M. (2015). One-step synthesis of amine-functionalized hollow mesoporous silica nanoparticles as efficient antibacterial and anticancer materials. ACS Appl. Mater. Interfaces.

[65] Zheng G., Shen Y., Zhao R., Chen F., Zhang Y., Xu A., Shao J. (2017). Dual-Targeting Multifuntional Mesoporous Silica Nanocarrier for Codelivery of siRNA and Ursolic Acid to Folate Receptor Overexpressing Cancer Cells. J. Agric. Food Chem..

[66] Cheng W., Liang C., Wang X., Tsai H.-I., Liu G., Peng Y., Nie J., Huang L., Mei L., Zeng X. (2017). A drug-self-gated and tumor microenvironment-responsive mesoporous silica vehicle: “four-in-one” versatile nanomedicine for targeted multidrug-resistant cancer therapy. Nanoscale.

[67] Xiao D., Hu J.-J., Zhu J.-Y., Wang S.-B., Zhuo R.-X., Zhang X.-Z. (2016). A redox-responsive mesoporous silica nanoparticle with a therapeutic peptide shell for tumor targeting synergistic therapy. Nanoscale.

[68] Höltke C. (2018). isoDGR-Peptides for Integrin Targeting: Is the Time Up for RGD?. J. Med. Chem..

[69] Meng F., Liu J., Wei J., Yang J., Zhou C., Yan J., Liu B. (2022). Tumor-penetrating peptide internalizing RGD enhances radiotherapy efficacy through reducing tumor hypoxia. Cancer Sci..

[70] Wang Y., Xie Y., Kilchrist K.V., Li J., Duvall C.L., Oupický D. (2020). Endosomolytic and Tumor-Penetrating Mesoporous Silica Nanoparticles for siRNA/miRNA Combination Cancer Therapy. ACS Appl. Mater. Interfaces.

[71] Ben-Uliel S.F., Zoabi F.H., Slavin M., Sibony-Benyamini H., Kalisman N., Qvit N. (2022). De Novo Development of Mitochondria-Targeted Molecular Probes Targeting Pink1. Int. J. Mol. Sci..

[72] Leserman L.D., Weinstein J.N., Blumenthal R., Terry W.D. (1980). Receptor-mediated endocytosis of antibody-opsonized liposomes by tumor cells. Proc. Natl. Acad. Sci. USA.

[73] Geles K.G., Gao Y., Giannakou A., Sridharan L., Yamin T.-T., Zhang J., Karim R., Bard J., Piche-Nicholas N., Charati M. (2021). NOTCH3-targeted antibody drug conjugates regress tumors by inducing apoptosis in receptor cells and through transendocytosis into ligand cells. Cell Rep. Med..

[74] Krishnamurti U., Silverman J.F. (2014). HER2 in breast cancer: A review and update. Adv. Anat. Pathol..

[75] Hapuarachchige S., Si G., Huang C.T., Lesniak W.G., Mease R.C., Guo X., Gabrielson K., Artemov D. (2021). Dual-Modality PET–SPECT Image-Guided Pretargeting Delivery in HER2(+) Breast Cancer Models. Biomacromolecules.

[76] Ngamcherdtrakul W., Sangvanich T., Reda M., Gu S., Bejan D., Yantasee W. (2018). Lyophilization and stability of antibody-conjugated mesoporous silica nanoparticle with cationic polymer and PEG for siRNA delivery. Int. J. Nanomed..

[77] Ngamcherdtrakul W., Bejan D.S., Cruz-Muñoz W., Reda M., Zaidan H.Y., Siriwon N., Marshall S., Wang R., Nelson M.A., Rehwaldt J.P. (2022). Targeted Nanoparticle for Co-delivery of HER2 siRNA and a Taxane to Mirror the Standard Treatment of HER2+ Breast Cancer: Efficacy in Breast Tumor and Brain Metastasis. Small.

[78] Iyer A.K., Khaled G., Fang J., Maeda H. (2006). Exploiting the enhanced permeability and retention effect for tumor targeting. Drug Discov. Today.

[79] Adams G.P., Schier R., Marshall K., Wolf E.J., McCall A.M., Marks J.D., Weiner L.M. (1998). Increased affinity leads to improved selective tumor delivery of single-chain Fv antibodies. Cancer Res..

[80] Rudnick S.I., Lou J., Shaller C.C., Tang Y., Klein-Szanto A.J., Weiner L.M., Marks J.D., Adams G.P. (2011). Influence of affinity and antigen internalization on the uptake and penetration of Anti-HER2 antibodies in solid tumors. Cancer Res..

[81] Fujimori K., Covell D.G., Fletcher J.E., Weinstein J.N. (1989). Modeling analysis of the global and microscopic distribution of immunoglobulin G, F(ab’)2, and Fab in tumors. Cancer Res..

[82] Schmidt M.M., Thurber G.M., Wittrup K.D. (2008). Kinetics of anti-carcinoembryonic antigen antibody internalization: Effects of affinity, bivalency, and stability. Cancer Immunol. Immunother..

[83] He F., Wen N., Xiao D., Yan J., Xiong H., Cai S., Liu Z., Liu Y. (2020). Aptamer-Based Targeted Drug Delivery Systems: Current Potential and Challenges. Curr. Med. Chem..

[84] Darmostuk M., Rimpelova S., Gbelcova H., Ruml T. (2015). Current approaches in SELEX: An update to aptamer selection technology. Biotechnol. Adv..

[85] Fu Z., Xiang J. (2020). Aptamers, the Nucleic Acid Antibodies, in Cancer Therapy. Int. J. Mol. Sci..

[86] Rabiee N., Ahmadi S., Arab Z., Bagherzadeh M., Safarkhani M., Nasseri B., Rabiee M., Tahriri M., Webster T.J., Tayebi L. (2020). Aptamer Hybrid Nanocomplexes as Targeting Components for Antibiotic/Gene Delivery Systems and Diagnostics: A Review. Int. J. Nanomed..

[87] Khatami F., Matin M.M., Danesh N.M., Bahrami A.R., Abnous K., Taghdisi S.M. (2021). Targeted delivery system using silica nanoparticles coated with chitosan and AS1411 for combination therapy of doxorubicin and antimiR-21. Carbohydr. Polym..

[88] Wan M., Chen H., Wang Q., Niu Q., Xu P., Yu Y., Zhu T., Mao C., Shen J. (2019). Bio-inspired nitric-oxide-driven nanomotor. Nat Commun..

[89] Marko A.J., Borah B.M., Siters K.E., Missert J.R., Gupta A., Pera P., Isaac-Lam M.F., Pandey R.K. (2020). Targeted Nanoparticles for Fluorescence Imaging of Folate Receptor Positive Tumors. Biomolecules.

[90] Chen Q., Meng X., McQuade P., Rubins D., Lin S.-A., Zeng Z., Haley H., Miller P., Trotter D.G., Low P.S. (2017). Folate-PEG-NOTA-Al^18^F: A New Folate Based Radiotracer for PET Imaging of Folate Receptor-Positive Tumors. Mol. Pharm..

[91] Cheng W., Nie J., Gao N., Liu G., Tao W., Xiao X., Jiang L., Liu Z., Zeng X., Mei L. (2017). A Multifunctional Nanoplatform against Multidrug Resistant Cancer: Merging the Best of Targeted Chemo/Gene/Photothermal Therapy. Adv. Funct. Mater..

[92] Huang G., Huang H. (2018). Application of hyaluronic acid as carriers in drug delivery. Drug Deliv..

[93] García-Posadas L., Contreras-Ruiz L., Arranz-Valsero I., López-García A., Calonge M., Diebold Y. (2014). CD44 and RHAMM hyaluronan receptors in human ocular surface inflammation. Graefe’s Arch. Clin. Exp. Ophthalmol..

[94] Mansoori-Kermani A., Khalighi S., Akbarzadeh I., Niavol F.R., Motasadizadeh H., Mahdieh A., Jahed V., Abdinezhad M., Rahbariasr N., Hosseini M. (2022). Engineered hyaluronic acid-decorated niosomal nanoparticles for controlled and targeted delivery of epirubicin to treat breast cancer. Mater. Today Bio.

[95] Ding J., Liang T., Zhou Y., He Z., Min Q., Jiang L., Zhu J. (2017). Hyaluronidase-triggered anticancer drug and siRNA delivery from cascaded targeting nanoparticles for drug-resistant breast cancer therapy. Nano Res..

[96] Shi X.-L., Li Y., Zhao L.-M., Su L.-W., Ding G. (2019). Delivery of MTH1 inhibitor (TH287) and MDR1 siRNA via hyaluronic acid-based mesoporous silica nanoparticles for oral cancers treatment. Colloids Surf. B Biointerfaces.

[97] Sanfilippo V., Caruso V.C.L., Cucci L.M., Inturri R., Vaccaro S., Satriano C. (2020). Hyaluronan-Metal Gold Nanoparticle Hybrids for Targeted Tumor Cell Therapy. Int. J. Mol. Sci..

[98] Du Rietz H., Hedlund H., Wilhelmson S., Nordenfelt P., Wittrup A. (2020). Imaging small molecule-induced endosomal escape of siRNA. Nat. Commun..

[99] Vermeulen L.M.P., De Smedt S.C., Remaut K., Braeckmans K. (2018). The proton sponge hypothesis: Fable or fact?. Eur. J. Pharm. Biopharm..

[100] Zhu J., Qiao M., Wang Q., Ye Y., Ba S., Ma J., Hu H., Zhao X., Chen D. (2018). Dual-responsive polyplexes with enhanced disassembly and endosomal escape for efficient delivery of siRNA. Biomaterials.

[101] Parnian J., Ma’mani L., Bakhtiari M.R., Safavi M. (2022). Overcoming the non-kinetic activity of EGFR1 using multi-functionalized mesoporous silica nanocarrier for in vitro delivery of siRNA. Sci. Rep..

[102] Berg K., Selbo P.K., Prasmickaite L., Tjelle T.E., Sandvig K., Moan J., Gaudernack G., Fodstad O., Kjølsrud S., Anholt H. (1999). Photochemical internalization: A novel technology for delivery of macromolecules into cytosol. Cancer Res..

[103] Zhang Y., Ren K., Zhang X., Chao Z., Yang Y., Ye D., Dai Z., Liu Y., Ju H. (2018). Photo-tearable tape close-wrapped upconversion nanocapsules for near-infrared modulated efficient siRNA delivery and therapy. Biomaterials.

[104] Jun E., Kim S., Kim J.-H., Cha K., So I.-S., Son H.-N., Lee B.-H., Kim K., Kwon I.C., Kim S.Y. (2015). Design of a multicomponent peptide-woven nanocomplex for delivery of siRNA. PLoS ONE.

[105] Yang Y., Sun B., Zuo S., Li X., Zhou S., Li L., Luo C., Liu H., Chen M., Wang Y. (2020). Trisulfide bond-mediated doxorubicin dimeric prodrug nanoassemblies with high drug loading, high self-assembly stability, and high tumor selectivity. Sci. Adv..

[106] Sun L., Liu Y.-J., Yang Z.-Z., Qi X.-R. (2015). Tumor specific delivery with redox-triggered mesoporous silica nanoparticles inducing neovascularization suppression and vascular normalization. RSC Adv..

[107] Pan Q.-S., Chen T.-T., Nie C.-P., Yi J.-T., Liu C., Hu Y.-L., Chu X. (2018). In Situ Synthesis of Ultrathin ZIF-8 Film-Coated MSNs for Codelivering Bcl 2 siRNA and Doxorubicin to Enhance Chemotherapeutic Efficacy in Drug-Resistant Cancer Cells. ACS Appl. Mater. Interfaces.

[108] Elmehrath S., Nguyen H.L., Karam S.M., Amin A., Greish Y.E. (2023). BioMOF-Based Anti-Cancer Drug Delivery Systems. Nanomaterials.

[109] Bäumer N., Tiemann J., Scheller A., Meyer T., Wittmann L., Suburu M.E.G., Greune L., Peipp M., Kellmann N., Gumnior A. (2022). Targeted siRNA nanocarrier: A platform technology for cancer treatment. Oncogene.

[110] Benassi E., Fan H., Sun Q., Dukenbayev K., Wang Q., Shaimoldina A., Tassanbiyeva A., Nurtay L., Nurkesh A., Kutzhanova A. (2021). Generation of particle assemblies mimicking enzymatic activity by processing of herbal food: The case of rhizoma polygonati and other natural ingredients in traditional Chinese medicine. Nanoscale Adv..

[111] Mathew B.T., Raji S., Dagher S., Hilal-Alnaqbi A., Mourad A.-H.I., Al-Zuhair S., Al Ahmad M., El-Tarabily K.A., Amin A. (2018). Bilirubin detoxification using different phytomaterials: Characterization and in vitro studies. Int. J. Nanomed..

[112] Contreras-Trujillo H., Eerdeng J., Akre S., Jiang D., Contreras J., Gala B., Vergel-Rodriguez M.C., Lee Y., Jorapur A., Andreasian A. (2021). Deciphering intratumoral heterogeneity using integrated clonal tracking and single-cell transcriptome analyses. Nat. Commun..

[113] Gao M., Chen Y., Wu C. (2021). Size-dependent chemosensitization of doxorubicin-loaded polymeric nanoparticles for malignant glioma chemotherapy. Bioengineered.

[114] Karai E., Szebényi K., Windt T., Fehér S., Szendi E., Dékay V., Vajdovich P., Szakács G., Füredi A. (2020). Celecoxib Prevents Doxorubicin-Induced Multidrug Resistance in Canine and Mouse Lymphoma Cell Lines. Cancers.

[115] Ward R.A., Fawell S., Floc’h N., Flemington V., McKerrecher D., Smith P.D. (2021). Challenges and Opportunities in Cancer Drug Resistance. Chem. Rev..

[116] Huang L., Zhao S., Fang F., Xu T., Lan M., Zhang J. (2021). Advances and perspectives in carrier-free nanodrugs for cancer chemo-monotherapy and combination therapy. Biomaterials.

[117] Ibrahim S., Baig B., Hisaindee S., Darwish H., Abdel-Ghany A., El-Maghraby H., Amin A., Greish Y. (2023). Development and Evaluation of Crocetin-Functionalized Pegylated Magnetite Nanoparticles for Hepatocellular Carcinoma. Molecules.

[118] Kumar P., Salve R., Paknikar K.M., Gajbhiye V. (2023). Nucleolin aptamer conjugated MSNPs-PLR-PEG multifunctional nanoconstructs for targeted co-delivery of anticancer drug and siRNA to counter drug resistance in TNBC. Int. J. Biol. Macromol..

[119] Wu H., Lv W.-H., Zhu Y.-Y., Jia Y.-Y., Nie F. (2023). Ultrasound-mediated mesoporous silica nanoparticles loaded with PDLIM5 siRNA inhibit gefitinib resistance in NSCLC cells by attenuating EMT. Eur. J. Pharm. Sci..

[120] Heidari R., Khosravian P., Mirzaei S.A., Elahian F. (2021). siRNA delivery using intelligent chitosan-capped mesoporous silica nanoparticles for overcoming multidrug resistance in malignant carcinoma cells. Sci. Rep..

[121] Song Y., Zhou B., Du X., Wang Y., Zhang J., Ai Y., Xia Z., Zhao G. (2020). Folic acid (FA)-conjugated mesoporous silica nanoparticles combined with MRP-1 siRNA improves the suppressive effects of myricetin on non-small cell lung cancer (NSCLC). Biomed. Pharmacother..

[122] Sun L., Wang D., Chen Y., Wang L., Huang P., Li Y., Liu Z., Yao H., Shi J. (2017). Core-shell hierarchical mesostructured silica nanoparticles for gene/chemo-synergetic stepwise therapy of multidrug-resistant cancer. Biomaterials.

[123] Subhan A., Attia S.A., Torchilin V.P. (2021). Advances in siRNA delivery strategies for the treatment of MDR cancer. Life Sci..

[124] Yuan Y., Liu J., Yu X., Liu X., Cheng Y., Zhou C., Li M., Shi L., Deng Y., Liu H. (2021). Tumor-targeting pH/redox dual-responsive nanosystem epigenetically reverses cancer drug resistance by co-delivering doxorubicin and GCN5 siRNA. Acta Biomater..

[125] Qi X., Yu D., Jia B., Jin C., Liu X., Zhao X., Zhang G. (2016). Targeting CD133(+) laryngeal carcinoma cells with chemotherapeutic drugs and siRNA against ABCG2 mediated by thermo/pH-sensitive mesoporous silica nanoparticles. Tumour Biol..

[126] Vecchio E., Golino G., Pisano A., Albano F., Falcone C., Ceglia S., Iaccino E., Mimmi S., Fiume G., Giurato G. (2019). IBTK contributes to B-cell lymphomagenesis in Eμ-myc transgenic mice conferring resistance to apoptosis. Cell Death Dis..

[127] Hou L., Song Z., Xu Z., Wu Y., Shi W. (2020). Folate-Mediated Targeted Delivery of siPLK1 by Leucine-Bearing Polyethylenimine. Int. J. Nanomed..

[128] Wen Y., Bai H., Zhu J., Song X., Tang G., Li J. (2020). A supramolecular platform for controlling and optimizing molecular architectures of siRNA targeted delivery vehicles. Sci. Adv..

[129] Phung C.D., Tran T.H., Choi J.Y., Jeong J.H., Ku S.K., Yong C.S., Kim J.O. (2021). Pre- and Post-Transcriptional Regulation of cFLIP for Effective Cancer Therapy Using pH-Ultrasensitive Nanoparticles. ACS Appl. Mater. Interfaces.

[130] Reda M., Ngamcherdtrakul W., Gu S., Bejan D.S., Siriwon N., Gray J.W., Yantasee W. (2019). PLK1 and EGFR targeted nanoparticle as a radiation sensitizer for non-small cell lung cancer. Cancer Lett..

[131] Popgeorgiev N., Sa J.D., Jabbour L., Banjara S., Nguyen T.T.M., Akhavan-E-Sabet A., Gadet R., Ralchev N., Manon S., Hinds M.G. (2020). Ancient and conserved functional interplay between Bcl-2 family proteins in the mitochondrial pathway of apoptosis. Sci. Adv..

[132] Juaid N., Amin A., Abdalla A., Reese K., Alamri Z., Moulay M., Abdu S., Miled N. (2021). Anti-Hepatocellular Carcinoma Biomolecules: Molecular Targets Insights. Int. J. Mol. Sci..

[133] Choi E., Lim D.-K., Kim S. (2020). Calcium-doped mesoporous silica nanoparticles as a lysosomolytic nanocarrier for amine-free loading and cytosolic delivery of siRNA. J. Ind. Eng. Chem..

[134] Punuch K., Wongwan C., Jantana S., Somboonyosdech C., Rodponthukwaji K., Kunwong N., Nguyen K.T., Sirivatanauksorn V., Sirivatanauksorn Y., Srisawat C. (2022). Study of siRNA Delivery via Polymeric Nanoparticles in Combination with Angiogenesis Inhibitor for The Treatment of *AFP*-Related Liver Cancer. Int. J. Mol. Sci..

[135] Li F., Wang Y., Chen W.-L., Wang D.-D., Zhou Y.-J., You B.-G., Liu Y., Qu C.-X., Yang S.-D., Chen M.-T. (2019). Co-delivery of VEGF siRNA and Etoposide for Enhanced Anti-angiogenesis and Anti-proliferation Effect *via* Multi-functional Nanoparticles for Orthotopic Non-Small Cell Lung Cancer Treatment. Theranostics.

[136] Abdalla A., Murali C., Amin A. (2022). Safranal Inhibits Angiogenesis via Targeting HIF-1α/VEGF Machinery: In Vitro and Ex Vivo Insights. Front. Oncol..

[137] Hamza A.A., Mohamed M.G., Lashin F.M., Amin A. (2020). Dandelion prevents liver fibrosis, inflammatory response, and oxidative stress in rats. J. Basic Appl. Zool..

[138] Gu H., Chen Y., Wang X., Liu T., Zhang D.S.-Z., Wang Y., Di W. (2015). Highly effective antiangiogenesis via magnetic mesoporous silica-based siRNA vehicle targeting the VEGF gene for orthotopic ovarian cancer therapy. Int. J. Nanomed..

[139] Ngamcherdtrakul W., Yantasee W. (2019). siRNA therapeutics for breast cancer: Recent efforts in targeting metastasis, drug resistance, and immune evasion. Transl. Res..

[140] Yang H., Yamazaki T., Pietrocola F., Zhou H., Zitvogel L., Ma Y., Kroemer G. (2015). STAT3 Inhibition Enhances the Therapeutic Efficacy of Immunogenic Chemotherapy by Stimulating Type 1 Interferon Production by Cancer Cells. Cancer Res..

[141] Iwata-Kajihara T., Sumimoto H., Kawamura N., Ueda R., Takahashi T., Mizuguchi H., Miyagishi M., Takeda K., Kawakami Y. (2011). Enhanced cancer immunotherapy using STAT3-depleted dendritic cells with high Th1-inducing ability and resistance to cancer cell-derived inhibitory factors. J. Immunol..

[142] Ngamcherdtrakul W., Reda M., Nelson M.A., Wang R., Zaidan H.Y., Bejan D.S., Hoang N.H., Lane R.S., Luoh S., Leachman S.A. (2021). In Situ Tumor Vaccination with Nanoparticle Co-Delivering CpG and STAT3 siRNA to Effectively Induce Whole-Body Antitumor Immune Response. Adv. Mater..

[143] Shahidi M., Abazari O., Dayati P., Bakhshi A., Zavarreza J., Modarresi M.H., Haghiralsadat F., Rahmanian M., Naghib S.M., Tofighi D. (2022). Multicomponent siRNA/miRNA-loaded modified mesoporous silica nanoparticles targeted bladder cancer for a highly effective combination therapy. Front. Bioeng. Biotechnol..

[144] Chen P., Wu Q., Feng J., Yan L., Sun Y., Liu S., Xiang Y., Zhang M., Pan T., Chen X. (2020). Erianin, a novel dibenzyl compound in Dendrobium extract, inhibits lung cancer cell growth and migration via calcium/calmodulin-dependent ferroptosis. Signal Transduct. Target. Ther..

[145] Han H.S., Choi K.Y. (2021). Advances in Nanomaterial-Mediated Photothermal Cancer Therapies: Toward Clinical Applications. Biomedicines.

